# Influence of Organismal Aging in Mesenchymal Stem Cell Therapy

**DOI:** 10.34297/ajbsr.2020.09.001363

**Published:** 2020-06-10

**Authors:** Arsalan Shabbir, Gen Suzuki, Techung Lee

**Affiliations:** Department of Biochemistry and Department of Medicine

## Introduction

Mesenchymal stem cells (MSCs) from several tissue sources have been investigated in clinical trials for multiple disorders, and mixed results from these trials have been documented [1], indicating that critical variables that can affect the therapeutic outcome remain to be defined. Organismal aging represents a potential impediment to stem cell therapy. Aged tissue often exhibits telomere shortening, increased Wnt signaling, and fibrosis [2,3], and may thus be more refractory to stem cell therapy. Accumulation of extracellular matrix (ECM) components, which invariably causes thickened lamina in aged tissue, can potentially impede the actions of the many growth/trophic factors secreted by the transplanted MSCs. Indeed, the aged heart often exhibits significant functional deteriorations contributed in part by cardiac stem cell senescence and lower capacity for angiogenesis [4]. Impaired HGF/c-Met and Delta/Notch signaling is also prominent in aged tissue [5].

**Figure 1:**
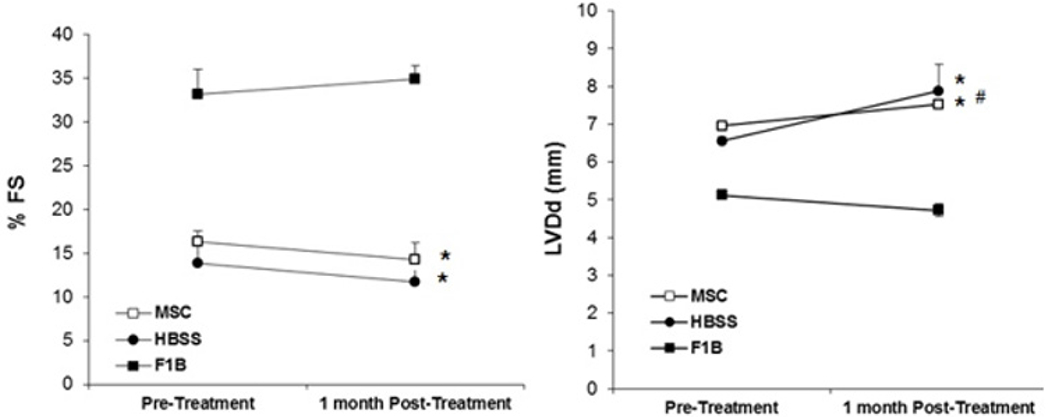
MSCs failed to improve cardiac function in old cardiomyopathic hamster. Ten-month-old F1B (normal) and TO_2_ hamsters were used for the study. TO_2_ hamsters received injections of Hanks Balanced Salt Solution (HBSS) or MSCs (n=5 per group). Echo measurements of %FS and LVDd were performed before injection and 1 month after injection. *p<0.05 vs. F1B; #p<0.05 vs. pre-injection.

This host tissue deficit remains a major challenge in regenerative medicine because the aging population usually require the therapy. We previously used a hamster (TO_2_ strain) heart failure model to study cardiac repair mechanisms mediated by MSCs [6]. However, these therapeutic studies were conducted using young animals (~4 months). Since the TO_2_ hamster heart is known to exhibit an early aging phenotype due to progressive loss of cardiomyocytes and functional decline, it is important to determine whether the aging heart of older TO2 hamster may be able to achieve functional improvement in response to MSC therapy. Echocardiography performed 1 month after MSC injection shows that both the saline (HBSS) - and MSC-treated old TO_2_ hamsters exhibited a similar decline in function as indicated by indistinguishable fractional shortening (FS) and left ventricular end-diastolic dimension (LVDd) between the two groups (Figure 1].

Thus, although MSC therapy is effective in treating younger TO_2_ hamsters, it is ineffective in treating the older cardiomyopathic hamsters. Advanced age typically exhibits more prevalent adverse events in humans. Although the human heart has been known to harbor a significant number of resident stem cells possessing limited regeneration capacity, age-related ECM remodeling and stem cell senescence can lead to declining cardiomyocyte populations and myocardial dysfunction [7,8]. Similar to our finding here, the cardiovascular beneficial effects of G-CSF and ischemic preconditioning were found to be impaired by aging [9,10]. Since the failing hamster heart exhibits abnormally active Wnt signaling-mediated fibrosis, excessive fibrosis in the 10-month TO_2_ heart may profoundly interfere with the growth factor signaling cascade mediated by the administered MSCs. The finding highlights the progressive nature of the fibrogenic process in the cardiomyopathic heart, which can interfere with the regenerative therapy. Thus, host tissue aging/fibrosis represents a major consideration in the design of MSC therapy.
